# Cloning of a novel thermostable glucoamylase from thermophilic fungus *Rhizomucor pusillus* and high-level co-expression with α-amylase in *Pichia pastoris*

**DOI:** 10.1186/s12896-014-0114-8

**Published:** 2014-12-24

**Authors:** Zhenggui He, Lujia Zhang, Youzhi Mao, Jingchao Gu, Qi Pan, Sixing Zhou, Bei Gao, Dongzhi Wei

**Affiliations:** State Key Laboratory of Bioreactor Engineering, New World Institute of Biotechnology, East China University of Science and Technology, Shanghai, 200237 China

**Keywords:** *Rhizomucor pusillus*, glucoamylase, α-amylase, Co-expression, *Pichia pastoris*

## Abstract

**Background:**

Fungal amylase, mainly constitute of fungal α-amylase and glucoamylase, are utilized in a broad range of industries, such as starch hydrolysis, food and brewing. Although various amylases have been found in fungi, the amylases from *Aspergillus* dominate the commercial application. One of main problems exist with regard to these commercial use of amylases is relatively low thermal and acid stability. In order to maximize the efficiency of starch process, developing fungal amylases with increased thermostability and acid stability has been attracting researchers’ interest continually. Besides, synergetic action of glucoamylase and α-amylase could facilitate the degradation of starch. And co-expressing glucoamylase with α-amylase in one host could avoid the need to ferment repeatedly and improves cost-effectiveness of the process.

**Results:**

A novel fungal glucoamylase (RpGla) gene encoding a putative protein of 512 amino acid residues was cloned from *Rhizomucor pusillus*. BLAST analysis revealed that RpGla shared highest identity of 51% with the *Rhizopus oryzae* glucoamylase (ABB77799.1). The fungal glucoamylase RpGla was expressed in *Pichia pastoris* (KM71/9KGla) with maximum activity of 1237 U ml^-1^. The optimum pH and temperature of RpGla were pH 4.0 and 70°C, respectively. Fungal α-amylase (RpAmy) gene was also cloned from *R. pusillus* and transformed into KM71/9KGla, resulted in recombinant yeast KM71/9KGla-ZαAmy harboring the RpGla and RpAmy genes simultaneously. The maximum saccharogenic activity of KM71/9KGla-ZαAmy was 2218 U ml^-1^, which improved 79% compared to KM71/9KGla. Soluble starch hydrolyzed by purified RpGla achieved 43% glucose and 34% maltose. Higher productivity was achieved with a final yield of 48% glucose and 47% maltose catalyzed by purified enzyme preparation produced by KM71/9KGla-ZαAmy.

**Conclusions:**

A novel fungal glucoamylase and fungal α-amylase genes were cloned from *Rhizomucor pusillus*. The two enzymes showed good thermostability and acid stability, and similar biochemical properties facilitated synergetic action of the two enzymes. A dramatic improvement was seen in amylase activity through co-expressing RpGla with RpAmy in *Pichia pastoris*. This is the first report of improving activity through co-expression glucoamylase with α-amylase in *P. pastoris*. Besides, fungal glucoamylase and α-amylase from *R. pusillus* were shown as promising candidates for further application in starch hydrolysis.

**Electronic supplementary material:**

The online version of this article (doi:10.1186/s12896-014-0114-8) contains supplementary material, which is available to authorized users.

## Background

Starch is one of the most abundant storage substances of plant. Amylases, which consist of multiple kinds of carbohydrase, hydrolyzing starch into diverse low molecular weight products, such as dextrin, maltose and glucose, are one of the most important enzymes [1,2]. Amylases are obtained from various sources including animals, plants and microorganisms, but only microbial amylase could meet the demands of industrial application due to its advantages in bulk production and other desired characteristics. Glucoamylase (EC 3.2.1.3) is an exo-acting enzyme which possesses strong activity of hydrolyzing polysaccharides into β-D-glucose by cleaving α-1, 4 glycosidic bond. Glucoamylase could also cleave α-1, 6 glycosidic bond, but at low rate. When at high concentration of glucose (35-40%), glucoamylase would catalyze glucose into oligosaccharides by reverse reaction [3]. Alpha-amylase (EC 3.2.1.1) is an endoamylase which hydrolyzes internal α-1, 4 glycosidic bond of starch, producing glucose, maltose and oligosaccharides [4].

Fungal amylase is more preferred in baking, brewing and sweeteners industries due to their more accepted generally recognized as safe status [5]. The processes of starch hydrolysis like saccharification need to perform at high temperature, however, the thermostabilities of most fungal glucoamylases are still not satisfactory. Therefore, developing fungal glucoamylase with increased thermostability especially at acidic condition will give an edge in the starch hydrolysis. To this, searching thermostable glucoamylase from thermophilic fungi provides a very attractive alternative. Although some thermostable glucoamylases from *Streptosporangium* sp*.* [6], *Aspergillus fumigates* [7,8] have been exploited, due to low yields and limited activity of these enzymes, nearly none of the thermostable fungal glucoamylase could meet the requirements of industrial production. So cloning the genes of thermostable fungal glucoamylase and heterologously expressing them in high-level expression system has been regarded as a promising way to accelerate the application of thermostable enzymes.

*Rhizomucor pusillus*, a thermophilic fungus, growing well even at 50°C, has been reported to be the source of amylase by Fergus [9] and Somkuti [10]. Deploey [11] later investigated the crude amylase produced by *Mucor pusillus* and found the optimal temperature and pH were 65°C and 4.5, respectively. And Silva *et al*. [12] studied the dextrinogenic and saccharogenic activity of crude amylase of *R. pusillus*. Although Kanlayakrit *et al*. [13] purified and characterized a raw-starch-digesting glucoamylase from *R. pusillus*, the gene encoding glucoamylase of *R. pusillus* has not been cloned thus far.

Co-expressing two or more enzymes in one host which avoids repeated fermentation facilitates the complex operation of extracting and purifying, and improves the cost-effectiveness of the process. It is an applicable and economic formulation in enzyme production. However, no report is presented on co-expressing glucoamylase with α-amylase in *Pichia pastoris*. Herein we reported on cloning a novel fungal glucoamylase and α-amylase from *R. pusillus* and high-level expression in *P. pastoris* respectively. Furthermore, we firstly co-expressed the two enzymes in *P. pastoris*, improving both saccharogenic activity and dextrinogenic activity significantly.

## Results and discussion

### Cloning and sequence analysis of RpGla and RpAmy genes

A partial gene fragments of 792-bp was amplified from genomic DNA of *R. pusillus* GX-3 with the degenerate primers. Then, a contiguous sequence extended to 3,200-bp (glu) was obtained by genome-walking method.

Using primers containing putative start codon or stop codon (designed according to the analysis of the sequence of glu), paired with primers corresponding to the conservative amino acids, several fragments were amplified from first-strand cDNA of *R. pusillus* GX-3. After sequencing and assembling the obtained cDNA fragments, one complete open reading frame (ORF) was obtained, and then confirmed by continuous PCR amplification. The ORF of *R. pusillus* glucoamylase (named as RpGla) consisted of 1539-bp nucleotides, encoding a putative protein of 512 amino acid residues. BLAST analysis revealed that RpGla shared the highest identity of 51% with the *Rhizopus oryzae* glucoamylase (ABB77799.1), indicating that RpGla was a novel glucoamylase. And the similarity with glucoamylse from *Mucor circinelloides* (AAN85206.1) and *Aspergillus niger* (AAT67041.1) were 50%, 36% respectively.

The RpGla was the second glucoamylase cloned from *Rhizomucor* sp.. Pedersen *et al*. [14] cloned the first glucoamylase gene from *Rhizomucor* sp. (*Mucor circinelloides*) for study of expression system, while did not investigate the enzymatic characterization.

RACE is the most widely used method to clone the full length cDNA sequence of eukaryotic gene, but it is costly and requires multiple steps. In this report, we tried to find the putative start codons and stop codons using the conserved sites as reference points. This successful attempt provided a helpful and effective method for cloning the cDNA of eukaryotic gene.

No signal peptide was present in the putative amino acid sequence of RpGla according to the analysis result using SignalP 4.0 program, implying that RpGla might be an intracellular or periplasmic enzyme.

The open reading frame of *R. pusillus* GX-3 α-amylase (RpAmy) was cloned and analyzed. It consisted of 1416-bp nucleotides, encoding a putative protein of 471 amino acid residues and had the highest similarity of 59% with the α-amylase from *Rhizopus oryzae* (ADL28123.1) [15].

### Transformation, screening of *Pichia pastoris* transformants

Increasing the copy number of expression cassette generally has the effect of increasing the amount of protein expressed in *P. pastoris* [16]. Transformants containing multiple copies of genes were obtained by electrotransformation and then screened on plates with higher concentrations of G418 or Zeocin. The colonies KM71/9KGla (RpGla expressed in *P. pastoris*) and KM71/ZαAmy (RpAmy expressed in *P. pastoris*) which chosen from YPDS plates containing 4.0 mg ml^−1^ G418 and 200 μg ml^−1^ Zeocin respectively were inoculated on BMMY plates containing 2% (w/v) soluble starch for amylase activity detecting. After incubating for 2 days, the colonies transformed with glucoamylase or α-amylase gene all showed clearance zones, while the original *P. pastoris* showed no clearance zones. (Additional file 1: Figure S1). It was demonstrated that RpGla and RpAmy could be successfully expressed in *P. pastoris* and secreted into the medium.

Plasmid ZαAmy was then transformed into recombinant *P. pastoris* KM71/9KGla. Since the recombinant *P. pastoris* KM71/9KGla could not grow on Zeocin contained YPDS plates (Additional file 2: Figure S2), it was indicated that the colonies on YPDS plates containing Zeocin was transformed with pPICZαAmy successfully. The resulted recombinant strain containing both fungal glucoamylase and α-amylase was designated as KM71/9KGla-ZαAmy.

### High-level expression of glucoamylase and α-amylase in *P. pastoris*

**Figure 1:**
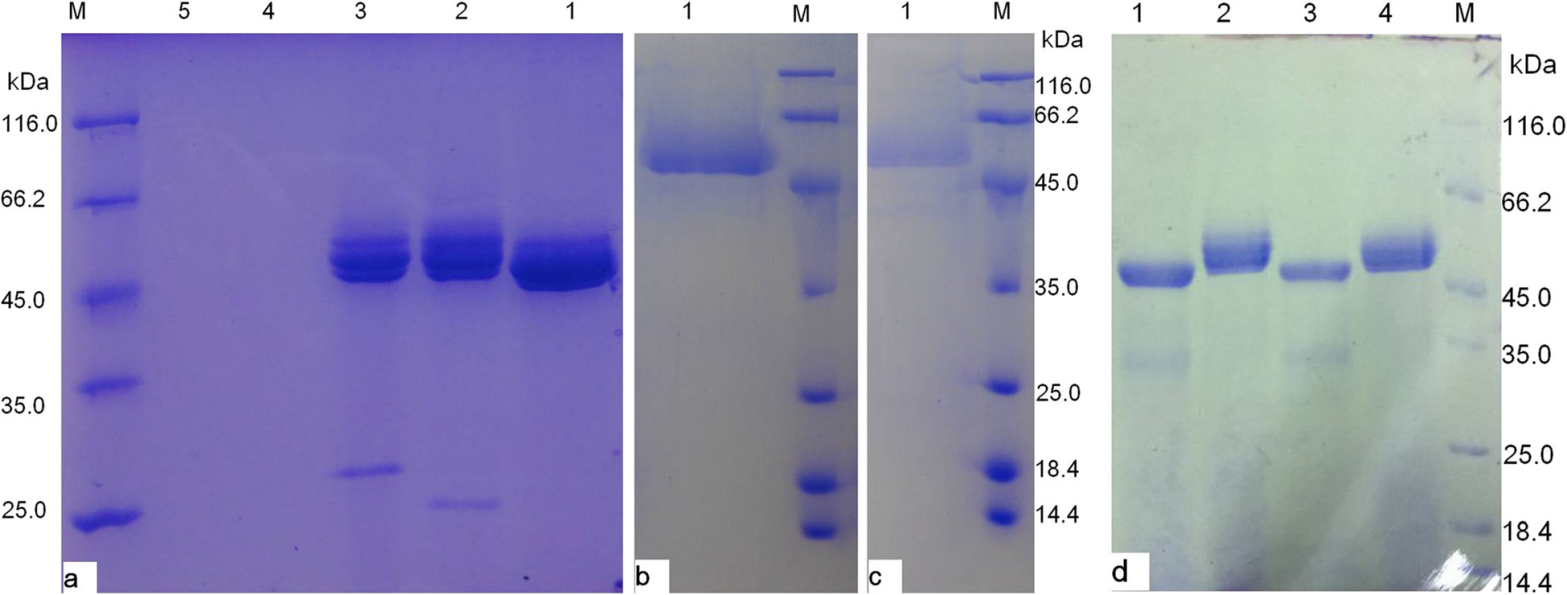
**SDS-PAGE analysis of recombinant RpGla and RpAmy expressed in recombinants** ***P. pastoris*** **KM71. (a)**: Lane 1. 10 μl of supernatant of recombinant KM71/9KGla-ZαAmy; Lane 2. 10 μl of supernatant of recombinant KM71/9KGla; Lane 3. 30 μl of supernant of recombinant KM71/ZαAmy; Lane 4. 10 μl of supernatant of recombinant transformed with empty vector pPIC9K; Lane 5. 10 μl of supernatant of recombinant transformed with empty vector pPICZα; Lane M. Molecular weight marker of proteins. **(b)**: Lane 1, 10 μl of recombinant RpGla obtained by partial purification; **(c)**: Lane 1, 10 μl of recombinant RpAmy obtained by partial purification. **(d)** Line 1, 10 μl of deglycosylated recombinant RpGla; Lane 2, 10 μl of recombinant RpGla; Line 3, 10 μl of deglycosylated recombinant RpAmy; Line 4, 10 μl of recombinant RpAmy.

**Figure 2:**
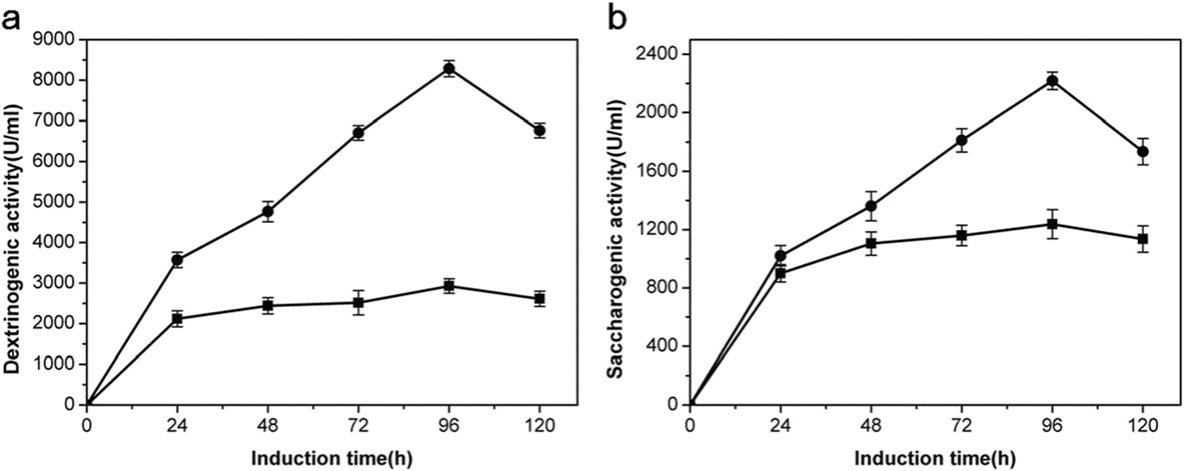
**Glucoamylase and α-amylase production of recombinant** ***P. pastoris*** **. (a)** Time course of saccharogenic activity of KM71/9KGla (■) and KM71/9KGla-ZαAmy (●); **(b)** Time course of dextrinogenic activity of KM71/ZαAmy (■) and KM71/9KGla-ZαAmy (●). The induction were performed in 500 ml shake flask with 50 ml BMMY media incubating in 30°C, 230 rpm. The induction period was 5 days with the addition of 0.5% (v/v) methanol per day.

After induction at 30°C, 230 rpm for 96 h, the supernatants of KM71/9KGla, KM71/ZαAmy and KM71/9KGla-ZαAmy all had a clear band between 45.0 kDa and 66.2 kDa (Figure 1a), while the supernatant of negative controls showed no band. The highest protein concentration of supernatant of KM71/9KGla-ZαAmy was 0.94 ± 0.13 mg ml^−1^, which was increased 23% compared to that of KM71/9KGla (0.76 ± 0.08 mg ml^−1^), and was greatly increased 298% compared to that of KM71/ZαAmy (0.24 ± 0.03 mg ml^−1^). The protein concentration of supernatant of negative controls were zero. Figure 2 illustrates the marked increase in activity. KM71/9KGla-ZαAmy achieved the highest saccharogenic activity (defined as the same with glucoamylase activity) of 2218 U ml^−1^, which was improved by 79% compared to that of KM71/9KGla (glucoamylase activity, 1237 U ml^−1^). The dextrinogenic activity (defined as the same with α-amylase activity) of KM71/9KGla-ZαAmy has a maximum activity of 8285 U ml^−1^ and was improved by 183% compared to that of KM71/ZαAmy (α-amylase activity, 2927 U ml^−1^). No amylase activity was detected in the original *P. pastoris* host.

Recently, special attention was paid to thermostable glucoamylase-producing fungi, but low yield and low activity have always been the bottleneck for their industrial application. The maximum activity of thermostable glucoamylases from *Streptosporangium* sp. [6] and *Humicola grisea var. ihermoidea* [17] and *Thermomyces lanuginosus* [18] were only 41 U ml^−1^, 15 U ml^−1^ and 1 U ml^−1^ respectively.

And the glucoamyalse and α-amylase activity of crude enzyme produced by *R. pusillus* GX-3 were 6.9 and 108 U ml^−1^ respectively. While the glucoamylase activity (saccharogenic activity) of KM71/9KGla and KM71/9KGla-ZαAmy increased by 179 and 321 times respectively compared to *R. pusillus* GX-3. It indicated that in our study, the two enzymes which heterologously expressed in *P. pastoris* showed potential application in starch degradation.

### Purification and properties of recombinant glucoamylase and α-amylase

The recombinant glucoamylase and α-amylase were purified by ammonium sulfate, and then submitted to SDS-PAGE analysis. The clear single band in Figure 1b and c indicated that the single step was sufficient for purification of obtained enzymes.

The recombinant RpGla was purified 1.2-fold with homogeneity and a recovery of 39%. The recombinant RpAmy was purified 1.7-fold with homogeneity and a recovery of 38%. The specific activity of purified RpGla and RpAmy were 1953 U mg^−1^ (glucoamylase activity) and 20732 U mg^−1^ (α-amylase activity).

Based on the gel filtration of purified recombinant RpGla (Additional file 3: Figure S3), there were two absorbance peaks corresponding to MW 114.5 kDa and 54.8 kDa respectively. While treated with boiling, the previous absorbance peak became smaller obviously (Additional file 3: Figure S3a). According to the SDS-PAGE analysis (Figure 1b), RpGla showed single band with estimated molecular weight of 45 ~ 66.2 kDa, and the theoretical molecular weight of RpGla was about 57 kDa. Therefore, the recombinant RpGla was inferred to be a dimer with a MW of 114.5 kDa.

The recombinant RpAmy showed the same phenomenon as RpGla. Two absorbance peaks corresponding to MW 114.9 kDa and 55.5 kDa respectively were presented (Additional file 3: Figure S3d). After boiled in water, the previous absorbance peak became much smaller (Additional file 3: Figure S3c). In view of the SDS-PAGE analysis, RpAmy showed single band with estimated molecular weight of 45 ~ 66.2 kDa, and the theoretical molecular weight of RpAmy was about 50 kDa. Therefore, the recombinant RpAmy was deduced to be a dimer with a MW of 114.9 kDa.

When submitted the translated amino acid sequence of RpGla and RpAmy to prediction of disulfide bond, little possibility of presence of disulfide bond was showed. So the dimer may be as a result of hydrophobic and polar interaction and be nonobligatory [19].

Lane 2 and Lane 4 in Figure 1d showed the deglycosylated recombinant RpAmy and RpGla respectively. The molecular weight of both recombinant proteins became a little smaller as a result of deglycosylation. According to the prediction results of glycosylation sites on NetNGlyc 1.0 (www.cbs.dtu.dk/services/), RpGla and RpAmy have 2 and 5 potential N-glycosylation sites respectively. So, RpGla and RpAmy expressed in *Phichia Pastoris* were glycoproteins.

**Figure 3:**
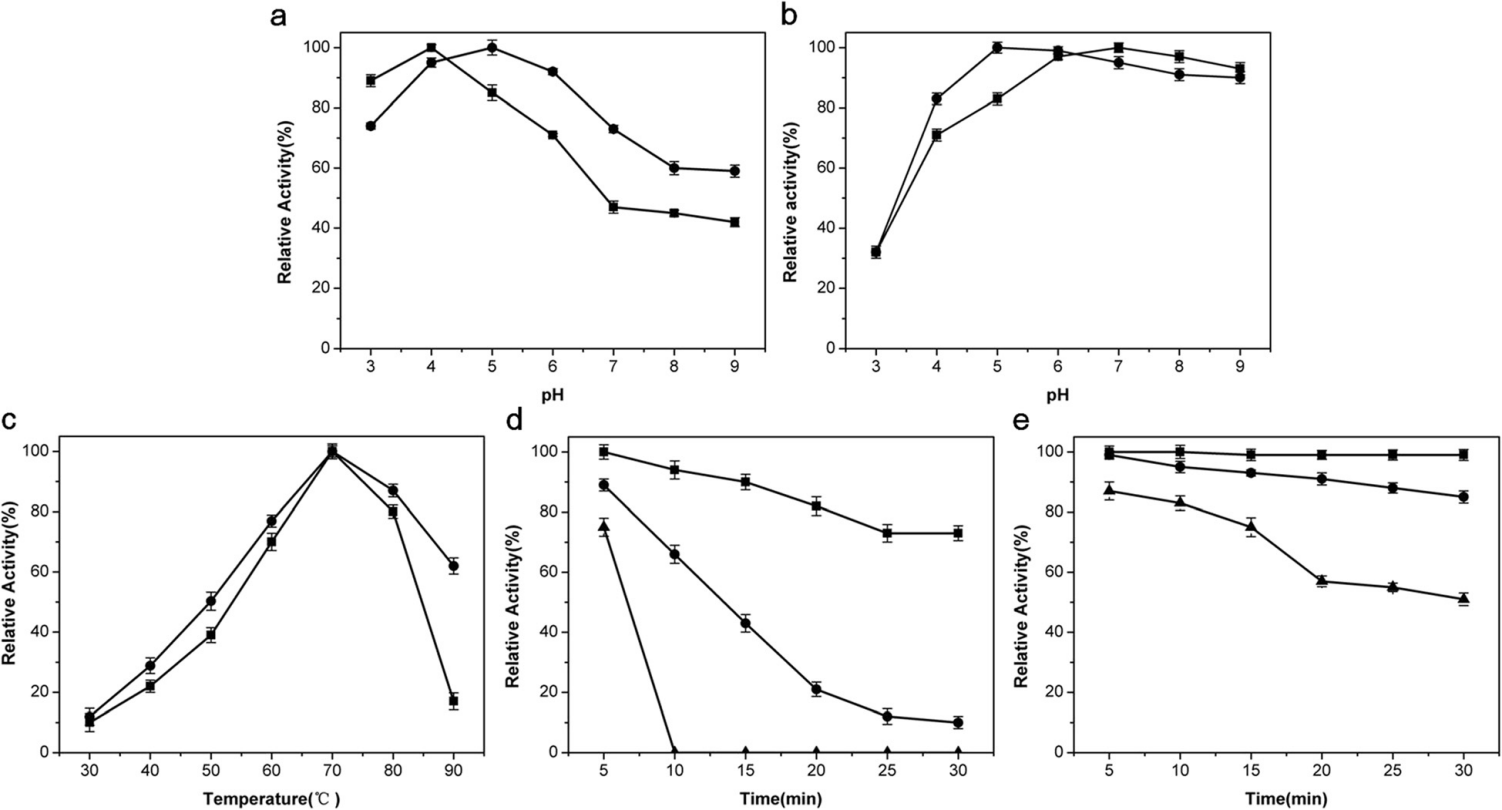
**Effect of pH and temperature on recombinant enzymes. (a)** Optimum pH of recombinant glucoamylase (■) and α-amylase (●); **(b)** pH stability of recombinant glucoamylase (■) and α-amylase (●); The optimal pH were studied between pH 3.0 and 9.0 at 60°C. The pH stability were determined after incubating enzymes at different pH buffers at 50°C for 30 min. **(c)** Optimum temperature of recombinant glucoamylase (■) and α-amylase (●); **(d)** Thermal stability of recombinant α-amylase (■60°C; ●70°C; ▲80°C); **(e)** Thermal stability of recombinant glucoamylase (■50°C; ●60°C; ▲70°C). The optimum temperature was monitored between 30°C and 90°C at pH 5.0. The thermostability was assayed after incubating the enzyme at different temperatures (50–80°C, pH 5.0). Samples were withdrawn per 5 minutes and placed on ice before the residual activities were assayed. All values were based on the average of triplicate measurements.

The RpGla and RpAmy both exhibited activity in acidic conditions (pH 3.0-6.0) with optimum at pH 4.0 and 5.0 (Figure 3a) respectively, and were stable in a wide range of pH 4.0-9.0 (Figure 3b). The optimal temperature for both enzymes was 70°C (Figure 3c). The RpGla and RpAmy showed relative good stability at 60°C. The residual activity of RpGla and RpAmy were 73% (Figure 3e) and 85% (Figure 3d), respectively, when incubated at 60°C for 30 min. The half-life (T1/2) of RpGla and RpAmy at 60°C were 164 min and 75 min respectively according to calculation based on kinetics of thermal deactivation of enzymes.

One of the main problems with regard to the industrial application of fungal glucoamylase is the relatively low thermal stability [20], like commercially available *Aspergillus niger* glucoamylase. Here, the thermal stabilities of RpGla and RpAmy were greatly higher than most other fungal amylases [1,3], making them a great potential for starch hydrolysis procedures.

Kanlayakrit *et al*. [13] have reported the purification and characterization of raw-starch-digesting glucoamylase from thermophilic *Rhizomucor pusillus*. The optimal temperature and pH were 65°C, 4.6 respectively, which were similar to the characteristics of RpGla in this work. However, in Kanlayakrit’s work, the activity of crude enzyme preparation was 17.7 U ml^−1^ and the specific activity was 57.7 U mg^−1^, which were both much lower than that of RpGla. Besides, as it is very common to have more than one glucoamylases in a fungus, thus it requires further study to explore whether the glucoamylase studied by Kanlayakrit was the same with RpGla.

Considering the similar biochemical properties of RpGla and RpAmy, one clear advantage was that the two enzymes could perform their best at the same condition. Consequently, we firstly co-expressed the glucoamylase with α-amylase in *P. pastoris* and exploit synergism in starch degradation. As described above, both saccharogenic activity and dextrinogenic activity were significantly enhanced by employing co-expression. Meanwhile, the excellent productivity of two enzymes in one host would provide cost reduction in fermentation, purification, and substantial savings in energy consumption. Note that the saccharogenic activity did not increase proportionally to the increase amount of recombinant α-amylase, it may ascribed to the fact that adding a few α-amylase into glucoamylase could facilitate the degradation of starch [21]. Since α-amylase hydrolyzes internal α-1, 4 glycosidic bond of starch randomly, providing more nonreducing chain ends, which could serve as substrate of glucoamylase, so the saccharogenic activity increased substantially.

Furthermore, according to the hydrolysis result (Table 1) of different starch catalyzed by the enzyme preparation, the saccharogenic activities towards all selected substrates were greatly enhanced by co-expression. Therefore, co-expression of the RpGla and RpAmy demonstrated advantages in liquefaction of different starch and other carbohydrate.

**Table 1:** **The hydrolysis of various substrates catalyzed by RpGla and RpAmy**

**Substrate**	**Relatively activity (%)**		
	**RpGla**	**RpAmy**	**Co-expression** ^**a**^
Soluble starch	55.8 ± 3.1	54.2 ± 2.7	100
maize starch	28.6 ± 0.8	34.9 ± 1.7	61.5 ± 2.8
potato starch	30.6 ± 1.8	28.8 ± 1.5	51.6 ± 2.4
amylopectin	66.4 ± 2.0	29.9 ± 0.7	79.2 ± 2.1
amylose	6.0 ± 0.2	5.2 ± 0.2	10.1 ± 0.4
glycogen	5.5 ± 0.2	5.8 ± 0.3	12.0 ± 0.5

^a^enzyme preparation produced by KM71/9KGla-ZαAmy. Substrates (0.05 g) in 0.1 M citric acid-sodium citrate buffer (pH 5.0, 5 ml) was mixed with 0.5 ml appropriately diluted enzyme. The reaction was carried out at 60°C for 10 min, and terminated by adding 1 ml of 3, 5-dinitrosalicylic acid and boiled for 5 minutes.

### Analysis of hydrolysis products

**Figure 4:**
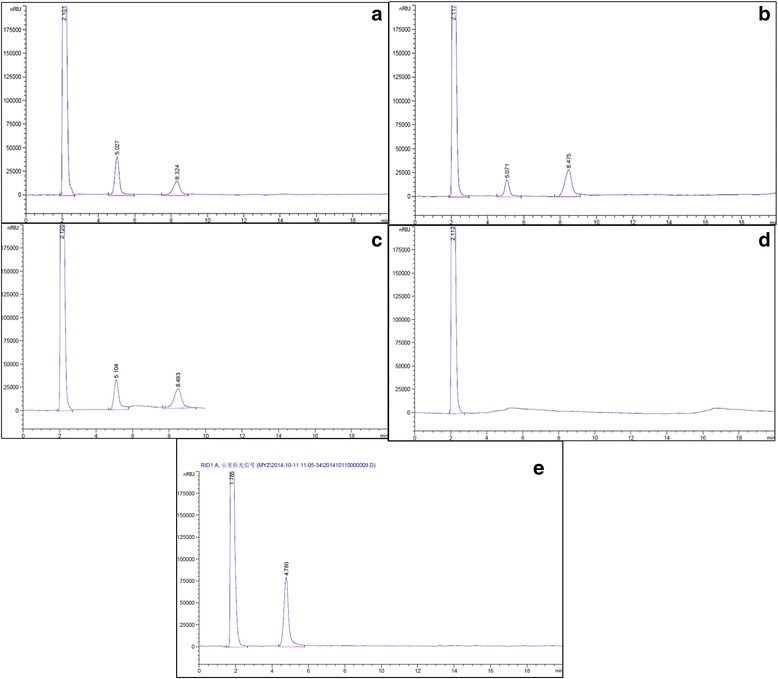
**HPLC analysis of catalyzed products of starch and glucose by recombinant enzymes. (a)** Hydrolysis products of starch catalyzed by purified RpGla; **(b)** Hydrolysis products of starch catalyzed by purified RpAmy; **(c)** Hydrolysis products of starch catalyzed by co-expressed enzyme preparation; **(d)** Blank control: soluble starch processed under the same procedure with deactivated enzyme preparation. The loading amount was all 10 μl. Substrate solution was prepared by mixing 1 g of soluble starch in 100 ml of citrate-sodium citrate buffer (50 mM, pH 5.0). 200 μl of appropriate diluted purified enzyme solution was added into 1 ml of substrate solution. After incubation at 50°C for 48 h, the mixture was boiled to stop the reaction and centrifuged. The retention time of glucose and maltose were about 5.0 min and 8.4 min respectively; **(e)** Reaction product of 40% glucose catalyzed by recombinant glucoamylase after 24 h of incubation at 50°C.

According to HPLC analysis result (Figure 4a), the main products of soluble starch hydrolyzed by purified RpGla were glucose (43%) and maltose (34%) with no detection of other oligosaccharides. Utilizing purified RpAmy as catalyst, the main products were maltose (69%) and glucose (22%) (Figure 4b) with no detectable maltotriose and maltotetraose. However, the main products hydrolyzed by purified mixed enzyme preparation of KM71/9KGla-ZαAmy were glucose (48%) and maltose (47%) with no detectable of maltotriose and maltotetraose (Figure 4c).

No extra peaks except glucose were detected up to 24 h of incubation even at high concentration (40%) (Figure 4d), which meant that the recombinant RpGla did not show any transglycosylation activity on glucose. Usually, transglycosylation activity of glucoamylase will lead to the formation of oligosaccharides from glucose with condensation reaction [22].

Therefore, the enhancement of hydrolysis reaction of recombinant glucoamyalse after adding recombinant α-amylases may owe to increased quantity of oligosaccharides and maltose that catalyzed by α-amylase [23].

## Conclusions

A novel fungal glucoamylase and the α-amylase genes were cloned from *Rhizomucor pusillus*, and expressed successfully in *Pichia pastoris*. The two recombinant enzymes have similar characteristics with optimal pH of 4.0 (RpGla) and 5.0 (RpAmy) respectively and optimal temperature as high as 70°C, thus the operating range in terms of pH, temperature and compatibility with other enzymes all improved. When co-expressing RpGla with RpAmy, the amylase activity improved significantly, and was much higher than most other thermostable fungal glucoamylases and α-amylases. The improved amylase activity through co-expressing glucoamylase with α-amylase in *P. pastoris* is first reported in this paper. Therefore, the work we did may offer an effective method to prepare high-activity biocatalyst. Meanwhile, the outstanding biochemical properties indicate emerging and promising application of *R. pusillus* glucoamyalse and α-amylase.

## Methods

### Strains, plasmids

*Rhizomucor pusillus* GX-3 was isolated in Henan, China. The strain grew well even at pH 4.0, 50°C. *Escherichia coli* DH5α and *Pichia pastoris* KM71 were used as hosts for DNA manipulation and gene expression. The vectors pMD19-T, pPIC9K and pPICZα were used for sequencing and expression of the amplified genes.

### Cloning and sequence analysis of glucoamylase and α-amylase

Preparation of genomic DNA was carried out according to the instructions of Fungal DNA Kit (Omega, USA). Total RNA was isolated from the mycelia of *R. pusillus* using RNAiso Plus (Takara). First-strand cDNA were synthesized using PrimeScript 1st Strand cDNA Synthesis Kit (Takara).

Two degenerated primers R.pGJf/R.pGJr (Table 2) corresponding respectively to the conserved catalytic domains WGRPQ(N)DGPA and GRYPED of glucoamylase amino acid sequence were designed to amplify fragments of the glucoamylase gene from *R. pusillus*. Genomic DNA and first-strand cDNA of *R. pusillus* GX-3 were used as templates respectively. The obtained DNA fragment was used as a starting template for chromosome-walking to obtain the full length of *R. pusillus* glucoamylase (RpGla). Subsequently, a contiguous sequence extended to 3,200 bp (glu) was obtained and confirmed by PCR amplification with primers R.pGWf/R.pGWr.

**Table 2:** **Primers used for PCR**

**Primers**	**Function**	**Sequence (5’-3’)**
R.pGJf	Primers for conserved regions of glucoamylase gene	TGGGGHMGHCCNCARAATGAYGG
R.pGJr		RTCGTCAGGRTANCKRCCRATDGC
5-1SP1	Primers for 5’-flanking region of glucoamylase gene	TCTTCCCTAGAATTGATGCGTGTGA
5-1SP2		AGTCTAAATCCTTGAATATCGCCGG
5-1SP3		AAGGATAAAGGTCGATGCACGCAGT
5-2SP1	Primers for 5’-flanking region of glucoamylase gene	ATCCTCCTCCTCCCATGAAGAAACA
5-2SP2		AGCAATATGTTGGTTGCGGTTGATC
5-2SP3		GAAAAGCATCAGCAGCACCTGAATC
3-1SP1	Primers for 3’-flanking region of glucoamylase gene	TGGATGTTTCTATCCTATTGGCAGC
3-2SP2		CCACATAAATATGGATCCATTGCCG
3-3SP3		TTGTATCCACTCAATCGGGAGCAGC
R.pGWr	Primers for glucoamylase gene glu’	GATGAAAGCAGCGTACGACCATGTC
R.pGWf		TGTGCAAGAATCTACCCTTTTCGAG
R.pGf1	Primers containing putative start codon for cDNA of glucoamylase gene	*ATG*CGTTATGCAACCCCGC
R.pGf2		*ATG*CTCTTCGCTTTTTGCTATTGT
R.pGf3		*ATG*TCTTACCGGAAGCAATTTCT
R.pGf4		*ATG*GGAGGAGGAGGATCTTGGT
R.pGr1	Primers containing putative stop codon for cDNA of glucoamylase gene	GGCG*TTA*TTTATTACCCTCTTTTGACC
R.pGf	Primers for cDNA of glucoamylase gene	A*TGG*ACA*CGC*GAT*TCA*GCCC
R.pGr		AGCGTACGACCATGTCAGGTCG
R.pGEcoRf	Primers containing restriction enzyme sites for cDNA of glucoamylase gene	G*GAATTC*ATGCGTTATGCAACCCCGC
R.pGNotr		TT*GCGGCCGC*TTACCCTCTTTTGACCA
R.pAf	Primers for cDNA of α-amylase gene	ATGAAATTCAGCATCTCTCTCTCGG
R.pAr		TTAAGCAGAGGTGAAGATAGCGGA
R.pAEf	Primers containing restriction enzyme sites for cDNA of α-amylase gene	GAATTCAGCCCTTTGCCCCAACAGCA
R.pANr		TTGCGGCCGCTTAAGCAGAGGTGAAGATAG

The primers are denoted as follows: the start codon and stop codon are underlined; the cons`ervative codons are boxed; the dotted line indicated restriction enzyme sites.

In order to clone the full length cDNA of glucoamylase, four primers (Table 2) containing putative start codon, one primer containing putative stop codon and two specific primers corresponding to the conservative amino acids (R.pGf, R.pGr) were designed based on the analysis of glu. The first-strand cDNA of *R. pusillus* GX-3 was used as template. The PCR products were sequenced and analyzed to reconstitute the putative open reading frame of *R. pusillus* glucomylase.

The cDNA of α-amylase gene was amplified with specific primers R.pAf and R.pAr (Table 2) using the first-strand cDNA of *R.pusillus* GX-3 as template. The signal peptides of deduced amino acid sequences were predicted on the SignalP 4.0 (www.cbs.dtu.dk/services/).

### Construction of the expression vectors and transformation of *P. pastoris*

The genes encoding the mature RpGla and RpAmy were amplified using primer pairs R.pGEf/R.pGNr and R.pAEf/R.pANr (Table 2), then cloned into pPIC9K and pPICZα, respectively. Recombinant vectors 9KGla and ZαAmy were transformed into *P. pastoris* KM71 by electrotransformation according to methods of Invitrogen manual, resulted transformants KM71/9KGla and KM71/ZαAmy, respectively. Transformants KM71/9KGla were seeded onto YPDS plates containing G418 at different concentration from 1 mg ml^−1^ to 4 mg ml^−1^ to screen multi-copy transfomants. Transformants KM71/ZαAmy were spread for selection on YPDS plates containing Zeocin of 100 μg ml^−1^, 200 μg ml^−1^.

In order to express glucoamylase and α-amylase simultaneously in *P. patoris*, plasmid ZαAmy was transformed into KM71/9KGla. The resulted recombinant containing both glucoamylase and α-amylase (designated as KM71/9KGla-ZαAmy) selected multi-copy clones by different concentration of Zeocin. The empty vectors pPIC9K and pPICZαA were transformed into KM71 as negative control.

### Expression and Purification of RpGla and RpAmy

Transformants KM71/9KGla and KM71/ZαAmy were inoculated on BMMY plates containing 2% (w/v) soluble starch to detect amylase activity. Recombinant *P. pastoris* which showed high amylase activity on BMMY plates were inoculated into 50 ml BMGY medium. The flasks were incubated in 30°C, 225 rpm. When OD_600_ reached 6.0, cells of 250 ml culture were harvested and then cultured in 50 ml BMMY media incubating in 30°C, 230 rpm to induce expression. The induction period was 5 days with the addition of 0.5% (v/v) methanol per day.

In order to test the production stabilities of the recombinant strain, more than three batches of production were performed at the same condition. The crude α-amylase in the supernatant was harvested by centrifugation. Solid ammonium sulphate was added into the crude supernatant (100 ml) up to 30% saturation, and the precipitated proteins were removed. Then adding solid ammonium sulphate up to 70% saturation, and collecting the precipitated recombinant enzyme. The precipitate was redissolved in 10 ml of 50 mM phosphate buffer (pH 6.0) and dialyzed overnight against the same buffer at 4°C. The dialysis was performed twice by renewing the buffer. The purified proteins were detected by SDS-PAGE, and the protein concentration were determined according to the method of Bradford [24]. All values are average values obtained from three independent experiments.

The molecular weight of the purified enzymes was estimated by gel filtration using Superdex 200 10/300 GL(GE Healthcare)equilibrated and eluted with 50 mM phosphate buffer plus 0.15 M NaCl, pH 6.5. The standard proteins (Thyroglobulin bovine, MW ~ 670 000, γ-globulins from bovine blood, MW ~ 150 000, Albumin chicken egg grade VI, MW ~ 44 300, Ribonuclease A type I-A from bovine pancreas, MW ~ 13 700) used as MW markers were purchased from Sigma. The loading sample was 100 μl. The experiment was conducted on ÄKTA™ system (GE Healthcare).

For the deglycosylation study, 10 μl purified recombinant RpGla or RpAmy were treated with 2 mU of Glycopeptidase F (Takara Biotechnology Co. Ltd, Dalian, China) for 17 h at 37°C according to the manufacturer’s instructions. The samples were run in SDS-PAGE for analysis.

### Enzyme assay

The glucoamylase activity or saccharogenic activity was estimated on the basis of increase in reducing sugars [25]. Soluble starch (0.05 g) in 0.1 M citric acid-sodium citrate buffer (pH 5.0, 5 ml) was mixed with 0.5 ml appropriately diluted enzyme. The reaction was carried out at 60°C for 10 min, and terminated by adding 1 ml of 3, 5-dinitrosalicylic acid and boiled for 5 minutes. One unit of glucoamylase activity or saccharogenic activity was defined as the amount of enzyme that released reducing sugars equivalent to 1 μmol of glucose per minute under the above conditions.

The activity of α-amylase activity or dextrinogenic activity was determined according to method described by Liu and Xu [26,27]. 0.5 ml of appropriately diluted enzyme and 5 ml of 0.5% (w/v) soluble starch dissolved in 0.1 M citric acid-sodium citrate buffer (pH 5.0) was incubated at 60°C. The reaction was terminated by the addition of 5 ml of chilled 0.1 M HCl. One unit of α-amylase activity or dextrinogenic activity was defined as the amount of enzyme that hydrolysis 1 mg of soluble starch in 5 minutes under the above conditions.

Maize starch, potato starch, amylopectin, amylose and glycogen were used to explore the extensive applicability of the synergic action of RpGla and RpAmy. The reactions were carried out as detection of saccharogenic activity [25]. The soluble starch catalyzed by purified enzyme preparation produced by KM71/9KGla-ZαAmy was regarded as control. The results were assayed and expressed as a percentage of the control values. All values were based on the average of triplicate measurements.

### Characterization of RpGla and RpAmy

The optimal pH of purified RpGla and RpAmy were studied between pH 3.0 and 9.0 (100 mM Gly-HCl buffer for pH 3.0, 100 mM citric acid-sodium citrate buffer for pH 4.0-5.0, 100 mM sodium phosphate buffer for pH 6.0-7.0, 100 mM Tris–HCl for pH 8.0-9.0) at 60°C. The pH stability profiles were determined by assaying the residual activity after incubating enzymes at different pH buffers at 50°C for 30 min. The optimum temperature of purified RpGla and RpAmy were monitored between 30°C and 90°C at pH 5.0. To test the thermostability, enzymes were incubated at different temperatures (50–70°C, pH 5.0, 100 mM citric acid-sodium citrate buffer). Samples were withdrawn per 5 minutes and placed on ice before the residual activities were assayed. All values were based on the average of triplicate measurements.

In order to investigate the hydrolysis product of starch catalyzed by purified RpGla and RpAmy, substrate solution was prepared by mixing 1 g of soluble starch in 100 ml of citrate-sodium citrate buffer (50 mM, pH 5.0). 200 μl of appropriate diluted purified enzyme solution was added into 1 ml of substrate solution. After incubation at 50°C for 48 h, the mixture was boiled to stop the reaction and centrifuged. The supernatant subjected to HPLC analysis. HPLC was performed on series 1200 HPLC system (Agilent Technologies, USA) using NH_2_- HPLC column (3.5 um, 3.0 × 250 mm) from Waters eluting with acetonitrile/water (85:15) at a flow rate of 0.8 ml min^−1^.

To check whether the recombinant RpGla has transglycosylation activity, the enzyme was incubated with different glucose concentrations (10, 20, and 40%) under pH 5.0 and 50°C. After incubation for 6, 12, 24 h, the reaction mixture was boiled to stop the reaction and centrifuged and then subjected to HPLC analysis for oligosaccharides detection [22].

The data sets supporting the results of this article are included within the article and its additional files.

### Nucleotide sequence accession numbers

The mRNA of *R. pusillus* glucoamylase and α-amylase had been submitted into the GenBank database under the accession numbers KC479790 and KC479791 respectively.

## Additional files

Additional file 1: Figure S1. Screening and detecting amylase activity of recombinant *P. pastoris* on BMMY plates containing 2% soluble starch. (a) KM71/9KGla; (b) KM71/ZαAmy; (c) *P. pastoris* KM71 transformed with empty vector pPIC9K or pPICZα. Additional file 2: Figure S2. Screening transformants KM71/9KGla-ZαAmy on YPDS plates containing 200 μg ml^−1^ Zeocin. (a) Recombinants KM71/9KGla inoculated on YPDS plates containing 200 μg ml^−1^ Zeocin; (b) Recombinants KM71/9KGla transformed with plasmid ZαAmy inoculated on YPDS plates containing 200 μg ml^−1^ Zeocin. Additional file 3: Figure S3. Elution profiles of boiled recombinant RpGla(a), recombinant RpGla(b), boiled recombinant RpAmy(c) and recombinant RpAmy. The gel filtration was conducted on ÄKTA™ system (GE Healthcare) with Superdex 200 10/300 GL(GE Healthcare)equilibrated and eluted with 50 mM phosphate buffer plus 0.15 M NaCl, pH 6.5. The flow rate was 0.5 ml/min. The standard proteins (Thyroglobulin bovine, MW ~ 670 000,γ-globulins from bovine blood, MW ~ 150 000, Albumin chicken egg grade VI, MW ~ 44 300, Ribonuclease A type I-A from bovine pancreas, MW ~ 13 700) used as MW markers were purchased from Sigma. The loading sample was 100 μl. The two absorbance peaks were corresponding to MW about 114 kDa and 55 kDa sequentially.
