# Supplementary figures and images for: Cloning of a novel thermostable glucoamylase from thermophilic fungus Rhizomucor pusillus and high-level co-expression with α-amylase in Pichia pastoris
Source: BMC Biotechnol. 2014 Dec 24;14:114. doi: 10.1186/s12896-014-0114-8 (PMC4362842; doi:10.1186/s12896-014-0114-8)

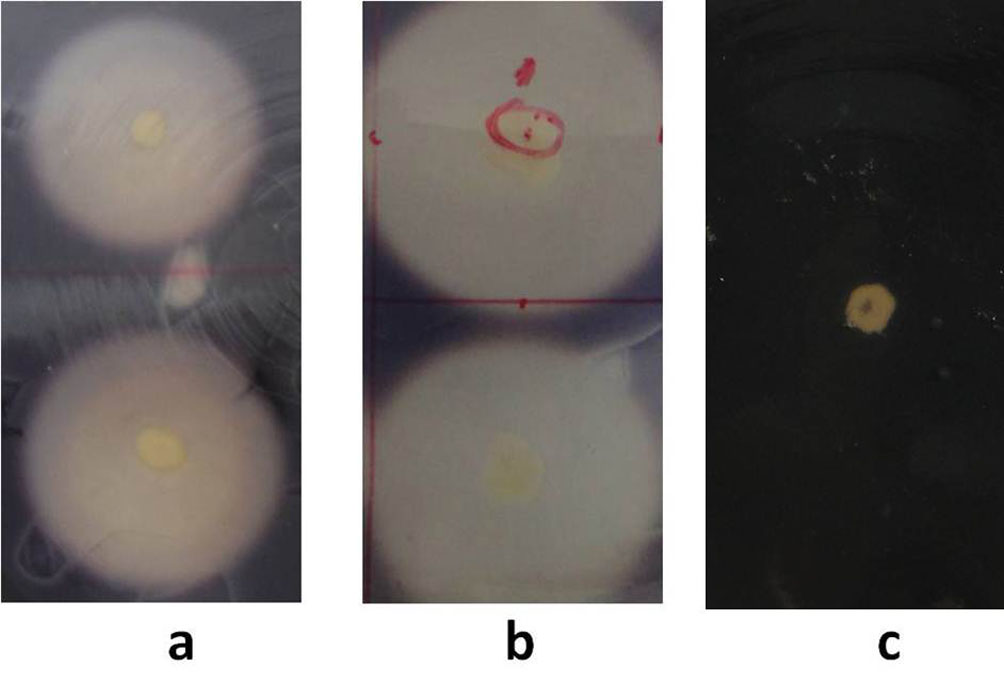

Supplement: Additional file 1: Figure S1. — Screening and detecting amylase activity of recombinant P. pastoris on BMMY plates containing 2% soluble starch. (a) KM71/9KGla; (b) KM71/ZαAmy; (c) P. pastoris KM71 transformed with empty vector pPIC9K or pPICZα. [file 12896_2014_114_MOESM1_ESM.jpeg]

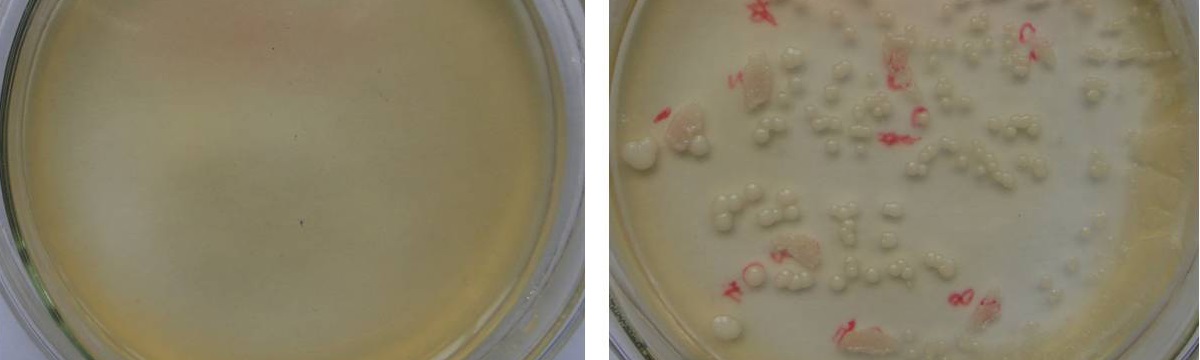

Supplement: Additional file 2: Figure S2. — Screening transformants KM71/9KGla-ZαAmy on YPDS plates containing 200 μg ml−1 Zeocin. (a) Recombinants KM71/9KGla inoculated on YPDS plates containing 200 μg ml−1 Zeocin; (b) Recombinants KM71/9KGla transformed with plasmid ZαAmy inoculated on YPDS plates containing 200 μg ml−1 Zeocin. [file 12896_2014_114_MOESM2_ESM.jpeg]

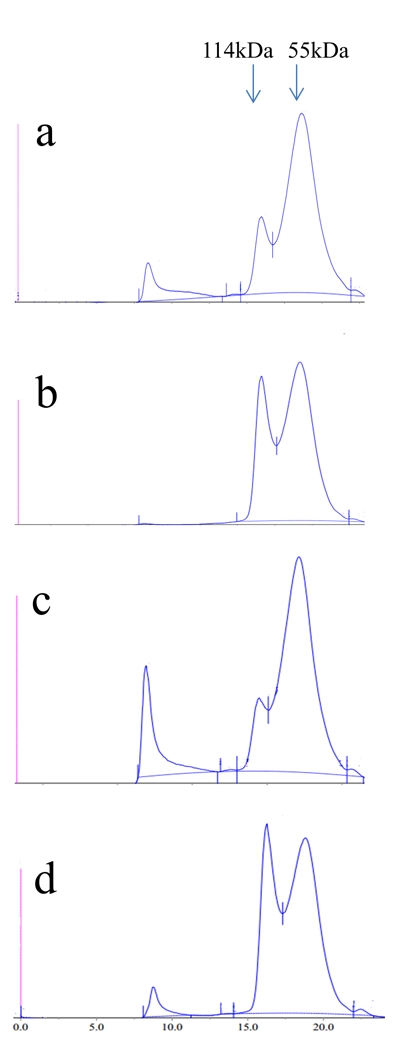

Supplement: Additional file 3: Figure S3. — Elution profiles of boiled recombinant RpGla(a), recombinant RpGla(b), boiled recombinant RpAmy(c) and recombinant RpAmy. The gel filtration was conducted on ÄKTA™ system (GE Healthcare) with Superdex 200 10/300 GL(GE Healthcare)equilibrated and eluted with 50 mM phosphate buffer plus 0.15 M NaCl, pH 6.5. The flow rate was 0.5 ml/min. The standard proteins (Thyroglobulin bovine, MW ~ 670 000,γ-globulins from bovine blood, MW ~ 150 000, Albumin chicken egg grade VI, MW ~ 44 300, Ribonuclease A type I-A from bovine pancreas, MW ~ 13 700) used as MW markers were purchased from Sigma. The loading sample was 100 μl. The two absorbance peaks were corresponding to MW about 114 kDa and 55 kDa sequentially. [file 12896_2014_114_MOESM3_ESM.jpeg]
